# Multimodal Neuroimaging Predictors of Learning Performance of Sensorimotor Rhythm Up-Regulation Neurofeedback

**DOI:** 10.3389/fnins.2021.699999

**Published:** 2021-07-20

**Authors:** Linling Li, Yinxue Wang, Yixuan Zeng, Shaohui Hou, Gan Huang, Li Zhang, Nan Yan, Lijie Ren, Zhiguo Zhang

**Affiliations:** ^1^School of Biomedical Engineering, Health Science Center, Shenzhen University, Shenzhen, China; ^2^Guangdong Provincial Key Laboratory of Biomedical Measurements and Ultrasound Imaging, Shenzhen, China; ^3^Marshall Laboratory of Biomedical Engineering, Shenzhen University, Shenzhen, China; ^4^Department of Neurology, Shenzhen Second People’s Hospital, The First Affiliated Hospital of Shenzhen University, Shenzhen, China; ^5^CAS Key Laboratory of Human-Machine Intelligence-Synergy Systems, Shenzhen Institutes of Advanced Technology, Chinese Academy of Sciences, Shenzhen, China; ^6^Peng Cheng Laboratory, Shenzhen, China

**Keywords:** neurofeedback (NFB), multimodal neuroimaging, sensorimotor rhythm (SMR), learning, functional connectivity (FC)

## Abstract

Electroencephalographic (EEG) neurofeedback (NFB) is a popular neuromodulation method to help one selectively enhance or inhibit his/her brain activities by means of real-time visual or auditory feedback of EEG signals. Sensory motor rhythm (SMR) NFB protocol has been applied to improve cognitive performance, but a large proportion of participants failed to self-regulate their brain activities and could not benefit from NFB training. Therefore, it is important to identify the neural predictors of SMR up-regulation NFB training performance for a better understanding the mechanisms of individual difference in SMR NFB. Twenty-seven healthy participants (12 males, age: 23.1 ± 2.36) were enrolled to complete three sessions of SMR up-regulation NFB training and collection of multimodal neuroimaging data [resting-state EEG, structural magnetic resonance imaging (MRI), and resting-state functional MRI (fMRI)]. Correlation analyses were performed between within-session NFB learning index and anatomical and functional brain features extracted from multimodal neuroimaging data, in order to identify the neuroanatomical and neurophysiological predictors for NFB learning performance. Lastly, machine learning models were trained to predict NFB learning performance using features from each modality as well as multimodal features. According to our results, most participants were able to successfully increase the SMR power and the NFB learning performance was significantly correlated with a set of neuroimaging features, including resting-state EEG powers, gray/white matter volumes from MRI, regional and functional connectivity (FC) of resting-state fMRI. Importantly, results of prediction analysis indicate that NFB learning index can be better predicted using multimodal features compared with features of single modality. In conclusion, this study highlights the importance of multimodal neuroimaging technique as a tool to explain the individual difference in within-session NFB learning performance, and could provide a theoretical framework for early identification of individuals who cannot benefit from NFB training.

## Introduction

Electroencephalographic (EEG) neurofeedback (NFB) training is a popular neuromodulation method to train brain functions. Through EEG NFB, one can learn to selectively enhance or inhibit his/her brain activities by means of real-time visual or auditory feedback of EEG signals (Sitaram et al., 2017). Since its first attempts in the 1960s, EEG NFB has rapidly received much attention because of its numerous potential applications for healthy participants and patients (Gruzelier, 2014a,b,c; Sitaram et al., 2017; Omejc et al., 2019). Among the diversity of NFB training protocols, one popular protocol is to up-regulate the amplitude of the sensory motor rhythm (SMR, 12-15 Hz), which is associated with a mental state of “relaxed alertness” (Witte et al., 2013). SMR NFB protocol has been applied to improve cognitive performance, such as sustained attention and visuo-motor skills of healthy participants (Gruzelier, 2014a; Kober et al., 2020). In clinical situation, SMR NFB training could improve the impaired cognitive functions in post-stroke patients (Kober et al., 2015). However, the efficacy of EEG NFB has been questioned recently because many sham-controlled studies have shown that a large proportion of users (16% to 57%) failed to self-regulate their brain activities and could not benefit from EEG NFB training (Gruzelier, 2014c; Thibault and Raz, 2016; Alkoby et al., 2018; Weber et al., 2020; Zhang et al., 2020a). It is of great importance to investigate the neural mechanisms of the huge individual differences in EEG NFB learning performance, because the successful learning during EEG NFB training can directly contribute to the improvement of disease symptoms in clinical patients (Gruzelier, 2014c). To identify in advance those participants who are likely to benefit from EEG NFB is a crucial step toward individualized and precise neuromodulation. Therefore, it is highly desirable to discover predictors of EEG NFB learning success and to establish a machine learning model to predict the learning performance based on identified predictors.

Some recent reviews concerned with the inefficiency problem of EEG NFB training have summarized various types of predictors of training performance (Alkoby et al., 2018; Weber et al., 2020). However, according to these reviews, only a very limited number of related studies have been carried out on and their findings are not convergent and complete. Standardized questionnaires or behavioral tasks are naturally considered as candidate predictors, but existing evidence showed that these questionnaires or tasks can predict NFB learning performance to a limited extend (Kleih et al., 2010; Nan et al., 2012; Witte et al., 2013; Alkoby et al., 2018). A batch of studies were focused on neurophysiological signals, mainly EEG recorded prior to training, for the prediction of performance during NFB training. In a resting-state EEG study, Nan et al. found that the amplitude of low beta (12–15 Hz) EEG rhythm measured before training could predict the NFB learning ability of low beta (15–18 Hz)/theta (4–7 Hz) ratio training (Nan et al., 2015). This research group later proposed that, eye-closed resting-state EEG activities in broad frequency bands, including lower alpha and theta, measured before training could distinguish learners/non-learners of alpha down-regulating NFB (Nan et al., 2018). Similarly, resting-state SMR power before training was related to the NFB training target at SMR activities (Reichert et al., 2015).

Besides above EEG predictors of NFB learning performance, magnetic resonance imaging (MRI) has also been more and more popularly used to investigate the problem of NFB inefficacy. because multimodal MRI can provide various types of predictors from brain structure, function, and connectivity with high spatial resolution. For example, structural MRI (sMRI) studies found that, the gray/white matter volume (GMV/WMV) could be the predictors of NFB performance and they were related to the neuroanatomical basis of the ability to learn to self-regulate one’s own brain activity (Weber et al., 2020). Resting-state functional MRI (rsfMRI) have also been applied to study neural mechanisms of individual differences in self-regulation of many other behaviors and brain functions (Kelley et al., 2015). Resting-state functional MRI also offers many metrices of local brain activity, such as regional homogeneity (ReHo) (Zang et al., 2004) and the amplitude of low-frequency fluctuations (ALFF) or fractional ALFF (fALFF) (Zou et al., 2008). All of this metrics can be used to investigated correlation between baseline brain activities and cognitive performance (Dong et al., 2015; Xiang et al., 2016; Xie et al., 2021). Particularly, because the interactions between brain regions are crucial for supporting cognitive functions, recent studies suggested that functional connectivity (FC) might be more promising for predicting complex high-order cognitive processes than those measured based on local brain regions (Qi et al., 2019; Horien et al., 2020). An fMRI-based NFB study suggested that rsfMRI FC can be used to identify individuals who are likely to benefit from fMRI NFB training to control anxiety symptom (Scheinost et al., 2014). However, rsfMRI (no matter which type of metrics) is still seldom used to investigate the inefficiency problem of SMR NFB.

In summary, the predictors of SMR up-regulation in NFB training has not been well understood yet. Multimodal brain imaging techniques could provide complementary and/or novel information about brain function, structure, and connectivity, so it is promising that more predictors could be discovered from multimodal brain imaging data and the accuracy to predict NFB learning performance can be improved. Thus, in the present study, we collected multimodal neuroimaging data (resting-state EEG, sMRI, and rsfMRI) before SMR-NFB experiments, with aims to identify the multimodal neuroanatomical and neurophysiological predictors for within-session learning performance of SMR up-regulation. The work could obtain an early identification of individuals who would not benefit from NFB training, and could increase the efficacy and cost-effectiveness of NFB technique in practical uses.

## Materials and Methods

### Participants

This study was approved by the Medical Ethics Committee of the Health Science Center of Shenzhen University. Thirty-four healthy participants (17 males, mean ± SD age: 22.8 ± 2.23) were recruited from Shenzhen University. The participants had no history of major medical illness, psychiatric or neurological disorder and had normal corrected-to-normal vision. All participants gave their written informed consent and the experimental procedure was approved by the local ethics committee.

### Data Acquisition

Electroencephalographic (EEG) signal during the NFB training was recorded with a 32-bit OpenBCI Board (Cyton Biosensing) connected to a lithium-ion polymer rechargeable battery. The OpenBCI board was connected to Ag/AgCl wet electrodes secured within an elastomeric EEG head-cap (Easy-Cap; Brain Products GmbH, Munich, Germany). Electrodes were placed at C1, C2, Cz, CPz, TP9, and TP10 positions according to the 10–20 electrode montage system. The reference electrode was located at FCz, and the ground electrode was located at the forehead.

Resting-state whole-brain EEG measurements (3-min eyes-open and eyes-closed) were also collected for each participant in a separate session before the first session of NFB training. EEG recordings were obtained with 64 Ag/AgCl electrodes placed on the EasyCap (Brain Products GmbH, Munich, Germany) with a reference electrode positioned at FCz. Vertical electrooculogram was recorded with the electrode placed on the superior to the nasion of the right eye. Input impedances were kept below 10 kΩ and the records were taken simultaneously at a sampling frequency of 1000 Hz.

Structural and resting-state functional MRI Images were collected using a 3.0 Tesla Siemens Trio scanner (Siemens Medical, Erlangen, Germany) at Shenzhen Institutes of Advanced Technology, Chinese Academy of Sciences. A standard 12-channel birdcage head coil was used and each participant’s head was fixed by foam pads in order to reduce head movement. Functional images were acquired with echo planar imaging sequence with the following parameters: 31 contiguous slices with a slice thickness of 4 mm; TR/TE = 2000/30 ms, 90° flip angle; field of view (FOV) 192 × 192 mm^2^; 64 × 64 data matrix. During the scanning of resting-state fMRI data, participants were asked to remain motionless, keep their eyes open, stay awake, and stare at the “+” sign. High resolution T1-weighted images were collected with a volumetric three-dimensional spoiled gradient recall sequence with the parameters: TR/TE = 2000/30 ms, FOV = 240 × 240 mm^2^, matrix size = 256 × 256, flip angle = 90°, slice number = 176, voxel size = 0.9 × 0.9 × 1 mm^3^.

### NFB Training

The SMR (12–15 Hz) up-regulating EEG NFB protocol was employed in this study, and each participant had to complete three sessions within one week and usually one day apart for continuous sessions. Each session consisted of 10 NFB training runs (3 min each). The first run is the baseline run during which the participants saw moving feedback bar but were instructed to relax themselves without trying to control the feedback bar voluntarily. The EEG signal recorded in the NFB baseline run was used to calculate initial individual threshold for SMR feedback and the threshold of SMR power was adapted after each run using the median value of SMR power in previous run.

During NFB training, the EEG signal was sampled at 250 Hz and band-pass filtered between 0.5 Hz and 45 Hz. The SMR power was calculated by fast Fourier-transforms (FFT) every 100 ms with a 10-s data window. The real-time SMR power (12–15 Hz) was presented on the screen in front of the participants as one vertical feedback bar. We only gave the participants a minimum degree of guidance, telling them to relax and concentrate physically during NFB training to increase the SMR power. When the SMR power exceeded the threshold, the color of the feedback bar changed from red to blue. Furthermore, when participants were able to move SMR feedback bars above the threshold and keeping for more than 1 s, they were rewarded with one more point displayed on the top of the screen.

The within-session NFB learning performance was assessed using a learning index (LI), which is calculated from the SMR powers of all NFB training runs and has been successfully applied in previous studies (Wan et al., 2014). Specifically, for each session, the median values of SMR power were calculated for all 12 training runs and then a linear regression was performed on these median values. Then LI was calculated as the average of the regression slopes across three sessions. The learning performance (LI) was checked for normality using Shapiro–Wilk test. It should be noted that the NFB learning index LI was calculated using SMR power values obtained from online EEG recorded by the OpenBCI cap during NFB training, because these on-line EEG results could better reflect the training performance.

### Analysis of Resting-State EEG

Resting-state EEG data recorded by the 64-channel EasyCap were analyzed by the Letswave toolbox^1^ and self-written MATLAB scripts. Continuous data were bandpass filtered between 1 Hz and 100 Hz. After visual inspection, bad channels were interpolated with adjacent channels. Then the signal was split into 1-s epochs and epochs with artefacts were rejected after visual inspection. These remaining epochs were submitted to an informax algorithm to decompose into their independent components (Makeig et al., 1997; Jung et al., 2001; Olbrich et al., 2011). The components related to eye blinking or movement were removed from the original data. Finally, all EEG signals were re-referenced to a common reference.

**FIGURE 1:**
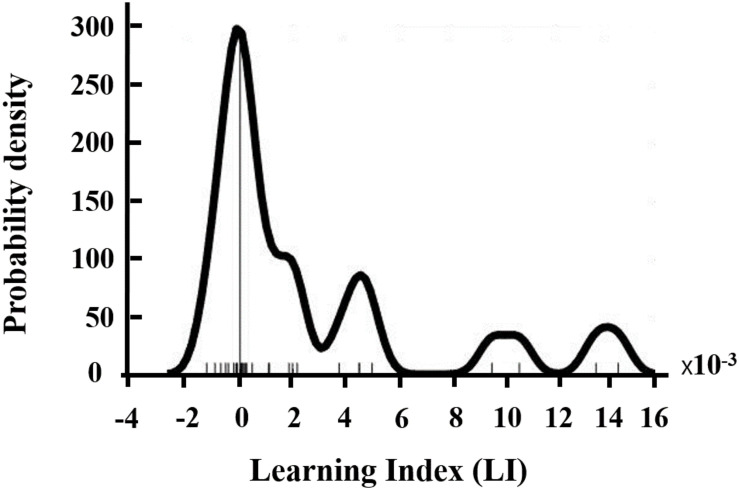
Distribution of NFB learning index LI.

After preprocessing, data were transformed into the frequency domain by the Welch’s method (with 1000-point FFT, 50% overlapping, and Hamming windows). Then we extracted the power values at four frequency bands: theta (4–8 Hz), alpha (8–12 Hz), SMR (12–15 Hz) and beta (12.5–30 Hz). Because EEG showed great inter-individual variabilities in the power, relative SMR power was calculated as the ratio of the power within a specific frequency band and the power and the total power in the full frequency band. For each NFB run, the relative power values were calculated for each epoch and then standardized across all epochs. Finally, the median value of relative powers of all epochs was taken as the relative power for the entire run. Furthermore, to assess the topological distribution of relative powers, nine scalp regions of interest (ROI) were defined (as shown in Figure 1) and they are: left frontal (F5, F3, FC5, FC3), middle frontal (F1, Fz, F2, FC1, FCz, FC2), right frontal (F4, F6, FC4, FC6), left central (C5, C3, CP5, CP3), middle central (C1, Cz, C2, CP1, CPz, CP2), right central (C4, C6, CP4, CP6), left parietal (P5, P3, PO7, PO3), middle parietal (P1, Pz, P2, POz), right parietal (P4, P6, PO4, PO8) (Reichert et al., 2015). The relative power of each ROI was calculated as the average across all the electrodes within this ROI.

Because the learning index (LI) of NFB training is not normally distributed (P = 0.0002), Spearman’s correlation analysis was performed to evaluate the correlation between resting-state EEG power and the learning performance (LI) of NFB training for each ROI at each frequency band. False discovery rate (FDR) correction was performed to address the multiple comparison problem (Benjamini and Hochberg, 1995).

### Analysis of Structural MRI Data

Voxel based morphometry analysis of sMRI images were performed using Computational Anatomy Toolbox^2^, which is an extension toolbox of Statistical Parametric Mapping (SPM12^3^). The sMRI images were segmented into gray matter, white matter and cerebrospinal fluid areas using the unified standard segmentation option in SPM12. The individual structural images were then normalized into standard Montreal Neurological Institute (MNI) template. Spatial normalization into the MNI standard space was done by the high-dimensional DARTEL (Diffeomorphic Anatomical Registration Through Exponentiated Lie Algebra) approach. Then the generated gray matter and white matter images were smoothed with an 8-mm full-width at half-maximum (FWHM) Gaussian kernel.

The voxel-wise statistical analyses were performed using statistical non-parametric mapping (SnPM) toolbox^4^. The non-parametric permutation approach was applied because it did not require any assumption on data normality (Nichols and Holmes, 2002). The standard general linear model (GLM) design setup was used by creating design matrices for multiple regression analysis of GMV or WMV, with individual NFB performance (LI), age and gender as regressors. General linear model was used to construct pseudo t-statistic images, which were then assessed for significance using a standard non-parametric multiple comparisons procedure based on randomization/permutation testing (*N* = 10000). Significant clusters were extracted with voxel-level *P* > 0.005 and cluster size >50.

### Analysis of Resting-State fMRI

The rsfMRI data were processed by using the Data Processing Assistant for Resting State fMRI (DPARSF^5^), which is based on SPM12 and the toolbox for Data Processing & Analysis of Brain Imaging (DPABI^6^). The preprocessing procedure was as follows. The first 10 volumes were removed to avoid T1 equilibration effects. Slice timing used the middle slice as the reference. Then the time series of rsfMRI images for each participant were realigned using a six-parameter (rigid body) linear transformation. The T1 images were co-registered to the mean functional image and then segmented into gray matter, white matter and cerebrospinal fluid. The Friston 24-parameter model was used to remove the linear trend and other nuisance signals (including Friston’s 24 motion parameters, cerebrospinal fluid, white matter). The rsfMRI data were then normalized to the MNI space and re-sampled to 3-mm isotropic voxels. A 6 mm FWHM Gaussian kernel were applied to smooth the rsfMRI data. Finally, a bandpass filter (0.01–0.1 Hz) was then performed on the rsfMRI data.

Three rsfMRI regional features, including ALFF, fALFF, and ReHo, were calculated. Amplitude of low-frequency fluctuations is the mean of amplitudes within a specific frequency domain of a voxel’s time course, and fALFF represents the relative contribution of specific oscillations to the whole detectable frequency range. ReHo is a rank-based Kendall’s coefficient of concordance (KCC) which shows the synchronization among a given voxel and its nearest neighbors (26 voxels) time courses. ALFF/fALFF was calculated using smoothed (unfiltered) rsfMRI timeseries, and ReHo was calculated using unsmoothed time series. The metric maps for ALFF/fALFF and ReHo were z-standardized (subtracting the mean value for the entire brain from each voxel, and dividing by the corresponding standard deviation). Normalized ReHo maps were then smoothed using a 6-mm FWHM.

The relationship between the learning index LI and three regional features were explored using SnPM toolbox. Standard GLM design setup was used by creating design matrices for multiple regression analysis of gray matter intensity, with individual NFB performance (learning index LI), age and gender as regressors. General linear model was used to construct pseudo t-statistic images, which were then assessed for significance using a standard non-parametric multiple comparisons procedure based on randomization/permutation testing (*N* = 10000). Significant clusters were extracted with voxel level *P* > 0.005 and cluster size >50.

Besides three regional rsfMRI features mentioned above, we also calculated the whole-brain FC using164 ROIs (160 ROIs from the Dosenbach atlas and four emotional related ROIs) (Dosenbach et al., 2010). The 164 ROIs were assigned into 7 intrinsic connectivity networks: (1) cerebellar, (2) cingulo-opercular, (3) default mode, (4) frontoparietal, (5) occipital, (6) sensorimotor, and (7) emotional. Each of the 164 ROIs was defined as a sphere with an 8 mm radius and the mean time series from all of the voxels within the ROI was extracted from preprocessed rsfMRI timeseries. We then calculated Pearson’s correlations between all pairs of ROIs for each participant to generate a 164 × 164 correlation matrix. The obtained correlation matrix for each participant was normalized using Fisher’s z-transformation.

Subsequently, we calculated the correlation between the learning index LI and rsfMRI FC strength between each ROI pairs, using the Spearman partial correlation analysis with age and gender as nuisance regressors. Significant resting-state FC were extracted with *P* < 0.005.

### Prediction of NFB Learning Performance

Lastly, machine learning models were trained to predict NFB learning performance (LI) from multimodal neuroimaging predictors (correlates), which were identified based on above mentioned analyses of multimodal neuroimaging data (Sections “Analysis of resting-state EEG,” “Analysis of structural MRI data,” and “Analysis of resting-state fMRI”). According to previous correlation analysis results between the NFB learning index and features of each imaging modality, four sets of features were employed in the prediction analysis: (1) band-limited relative power of resting-state EEG, (2) GMV/WMV of sMRI, (3) three regional features (ALFF, fALFF, ReHo) of rsfMRI, (4) FC of rsfMRI. To investigate whether the combination of multimodal features can improve the performance in predication of NFB learning index, we further compare the prediction performance using features from each modality as well as multimodal features. The set of multimodal features were constructed by concatenating all four types of feature vectors. Across-individual normalization was performed for each feature as well as the learning index LI before training the machine learning models.

Four machine learning techniques, namely, linear support vector regression (SVR), linear regression (LR), Bayesian automatic relevance determination regression (ARDR), and random forest regression (RFR), were used here to quantitatively predict the NFB learning index from multimodal neuroimaging features (Pereira et al., 2009). Note that, because there is only one EEG correlate (SMR power of the left central region) found to be significantly correlated with LI (see Section “EEG correlates of NFB learning performance”), a linear regression model was used instead of SVR to predict LI from this EEG predictor. A leave-one-out-cross-validation (LOOCV) strategy was adopted to evaluate the performance of the prediction models. For each iteration in LOOCV, one participant was selected as the test sample and fed to the linear regression model (for EEG) or the SVR model (for MRI and combined multimodal features) trained with remaining samples, and the iterations were repeated for each participant. To quantify the prediction performance, mean absolute error (MAE), which was calculated as the average of the absolute difference between actual and predicted values, as well as Pearson correlation between actual and predicted values were used. Furthermore, we compared the Pearson correlation coefficient obtained using multimodal features and the Pearson correlation coefficient obtained using each set of features from single modality. The calculation and comparison of Pearson correlations in this part were carried out using SPSS (SPSS Statistics, version 22, IBM, Armonk, NY).

## Results

### NFB Learning Performance

Seven participants were excluded from analysis due to incomplete training or excessive EEG artifacts. Therefore, data from 27 participants (12 males, age: 23.1 ± 2.36) were available for subsequent analysis. Most of the participants (19 of 27) were able to increase their SMR power within-session as suggested by their positive NFB learning index LI (as shown in Figure 1).

### EEG correlates of NFB Learning Performance

**FIGURE 2:**
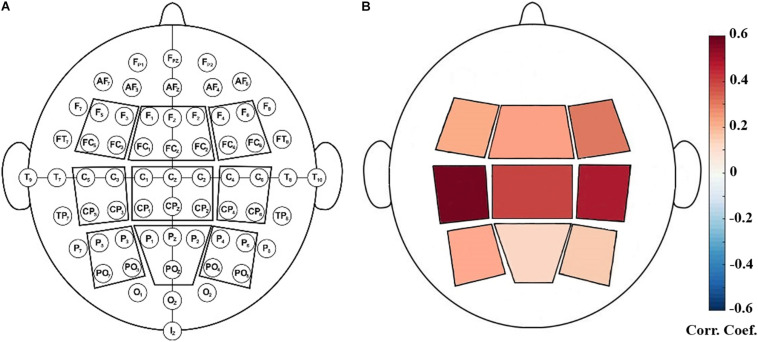
**(A)**: Illustration of defined EEG-scalp regions of interest (ROI). **(B)**: Positive correlation between SMR power at rest (baseline) and NFB learning index LI. The ROI outlined in red contained C5, C3, CP5, CP3 and it showed significant correlation with LI (*P* < 0.05, FDR-corrected).

We performed correlation analysis between the NFB learning index LI and EEG powers of nine scalp ROIs. As shown in Figure 2, LI had significant positive correlations with SMR power of left central ROI (including EEG channels C5, C3, CP5, and CP3) during the eyes-open resting-state conditions (*P* < 0.05, FDR-corrected).

### sMRI Correlates of NFB Learning Performance

**FIGURE 3:**
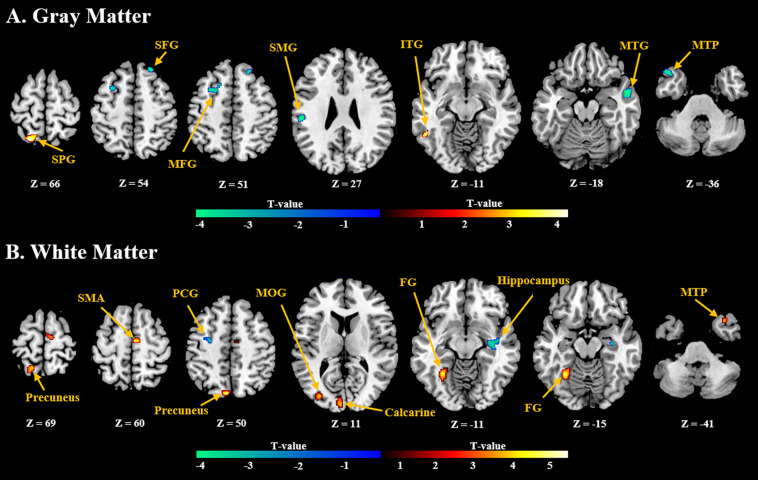
Brain regions with **(A)** gray and **(B)** white volumes which show significant positive (red) or negative (blue) correlation with the NFB learning index LI (*P* < 0.005, Cluster size > 50). SPG, Superior Parietal Gyrus; SFG, Superior Frontal Gyrus; MFG, Middle Frontal Gyrus; SMG, Supramarginal Gyrus; ITG, Inferior Temporal Gyrus; MTG, Middle Temporal Gyrus; MTP, Middle Temporal Pole; SMA, Supplementary Motor Area; PCG, Postcentral Gyrus; MOG, Middle Occipital Gyrus; FG, Fusiform Gyrus.

General linear model (GLM) analysis revealed that the NFB learning index LI was positively associated with GMV localized in the inferior temporal gyrus and superior parietal gyrus, and negatively associated with GMV localized in the frontal gyrus, middle temporal gyrus, and supramarginal gyrus (Figure 3A). For WMV, positive correlation was observed mainly in the supplementary motor area, precuneus, and medial occipitotemporal gyrus, and negative correlation was observed in the precentral gyrus and hippocampus (Figure 3B). Detailed information of these clusters with significant correlation can be found in Supplementary Table 1.

### rsfMRI Correlates of NFB Learning Performance

**FIGURE 4:**
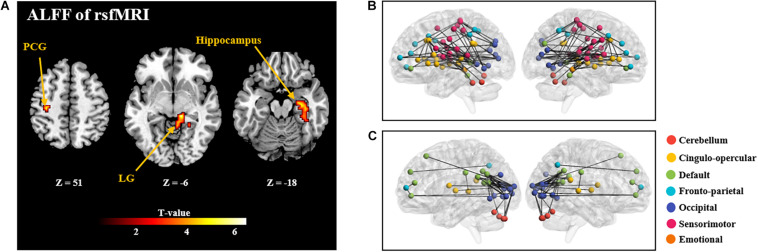
**(A)**: Brain regions with rsfMRI ALFF which are significantly correlated with the NFB learning index LI (*P* < 0.005, Cluster size > 50). PCG: Postcentral Gyrus; LG: Lingual Gyrus. **(B)** and **(C)** shows the correlation coefficients between rsfMRI FC and the NFB learning index LI (*P* < 0.005; **(B)**, positive correlations; **(C)**: negative correlations). In **B** and **C**, nodes of different colors represent different brain regions, and the edge thickness represents the FC strength.

GLM analysis found positive correlation between ALFF and NFB learning index LI in the postcentral gyrus, lingual gyrus, and hippocampus (as shown in Figure 4 and Supplementary Table 2). However, other two types of regional rsfMRI features (fALFF and ReHo) did not show any significant correlation results with LI. As a result, only the identified ALFF features were used to build a model for prediction of the NFB learning index.

Further, significant positive correlations between strength of rsfMRI FC and NFB learning index LI were mainly observed within the cingulo-opercular network, between the sensorimotor network and occipital network (*P* < 0.005; Figure 4B); and significant negative correlation was observed between the default mode network and occipital network (*P* < 0.005; Figure 4C).

### Prediction of NFB Learning Performance

**FIGURE 5:**
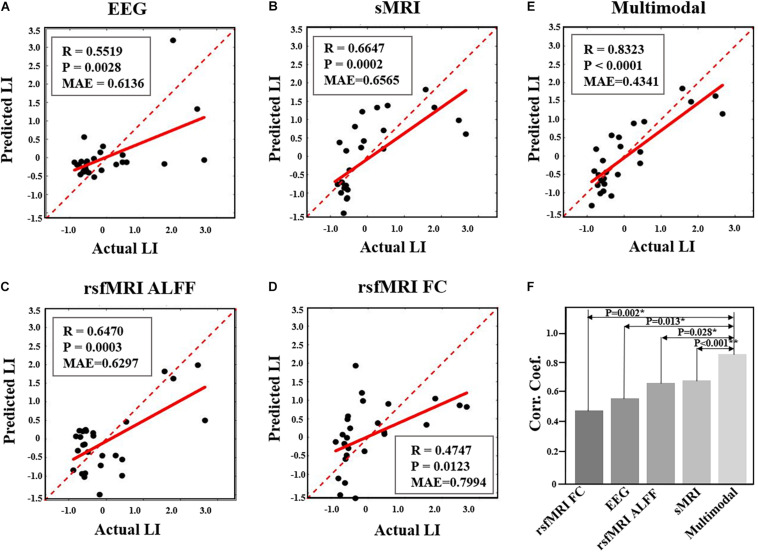
Comparison of performance in prediction NFB within-session learning index LI using different feature sets. **(A)**. Prediction results using EEG features. **(B)**. Prediction results using sMRI features. **(C)**. Prediction results using rsfMRI ALFF. **(D)**. Prediction results using rsfMRI FC. **(E)**. Prediction results using multimodal neuroimaging features. The red dashed lines represent y = x while the red solid lines represent the regression lines. Each black dot represents one participant. **(F)** shows the comparison of correlation coefficients between predicted and actual LI values among five different feature sets (*, *P* < 0.05; **, *P* < 0.005).

Prediction analysis of the NFB learning index LI was performed using features selected from each modality, as well as from multimodal features. According to the results using SVR model, the NFB learning index LI can be better predicted using multimodal neuroimaging features, as compared with using features of single modality (as shown in Figures 5). Specifically, the MAE of multimodal features is 0.4341, which is smaller than those of single modality (0.6136 for EEG features, 0.6565 for sMRI features, 0.6297 for rsfMRI ALFF features, 0.7994 for rsfMRI FC features) (Figures 5A–D). Moreover, the correlation coefficient between actual and predicted values using multimodal features are significantly higher than the correlation coefficients derived using features of single modality (Figure 5F). Prediction of NFB learning index LI using other models can be found in Supplementary Table 3 and Supplementary Figure 1, and generally better results could be obtained using multimodal neuroimaging features.

## Discussion

Investigating the neural correlates of an individual’s learning ability during NFB training is important since the learning ability has a crucial mediation link with the behavioral or clinical outcome after NFB training (Gruzelier, 2014a). Prediction of NFB learning performance would help prevent unnecessary time and resources used on participants who cannot learn to modulate their brain rhythms, and could make these participants choose other treatment means earlier. However, reliable predictors of NFB learning performance remain elusive. Thus, this study was aimed to investigate the association between learning performance during the SMR up-regulating NFB-training and multimodal neuroimaging data (resting-state EEG, sMRI, and rsfMRI), and then to assess whether NFB learning performance can be better predicted using multimodal features. According to our results, most participants were able to successfully increase the SMR power and the learning performance was significantly correlated with a set of EEG or MRI features. Importantly, results of prediction analysis indicate that NFB learning index can be better predicted using multimodal features compared with features of single modality. These results will be discussed in detail bellow.

### Advantages of Using Multimodal Data

Neurofeedback (NFB) training is generally based on real-time feedback of voluntarily induced changes of brain activities, and it is a process of operant conditioning which leads to self-regulation of brain activity. Successful self-regulation of brain activity during NFB training can be considered as a personal skill, and a relatively high proportion of participants cannot achieve stable self-regulation of the target brain activity (Alkoby et al., 2018). In line with the trend to study the relationship between an individual’s brain structure or function and individual differences in behavior, many NFB studies explained the individual differences of NFB learning performance based on neural recordings and brain imaging data, such as resting-state EEG features and neuroanatomical features (Alkoby et al., 2018; Weber et al., 2020). A number of EEG NFB studies suggested that participants can be grouped as “learners” or “non-learners,” based on their brain ability or inability to regulate their brain activity during the NFB training. Then, machine learning models can be trained to predict NFB learning performance using single-modality neuroimaging predictors (Wan et al., 2014; Nan et al., 2015; Reichert et al., 2015; Nan et al., 2018).

More and more studies used multimodal neuroimaging in human brain researches because it overcomes the limitations of individual modalities. Different neuroimaging techniques have different biochemical/biophysical mechanisms, which lead to different capabilities in probing the human brain’s structure and function (Zhang et al., 2020b). Specifically, EEG passively records electric changes induced by extra- and intra-cellular electric currents associated with neuronal brain activity (Michel and Murray, 2012). Electroencephalographic has a high temporal resolution and it is safe, cheap, and easy to operate. On the other hand, MRI has a good spatial resolution and can provide a more complete as well as more detailed picture of the brain. Structural MRI can provide static anatomical information (Symms et al., 2004) and fMRI depicts brain activity by detecting the changes in brain hemodynamics (Howseman and Bowtell, 1999). Multimodal neuroimaging data analysis could take the advantages from multiple imaging techniques, such as improving both spatial and temporal resolution and illustrating the anatomical basis for functional activities (Zhang et al., 2020b). Numerous neuroimaging studies have shown that, multiple neuroimaging modalities may provide a more comprehensive understanding of the complex interplay between the brain (including structure, function, and networks) and behavior. For example, integrating both functional and structural features could improve prediction accuracy of intelligence of healthy subjects (Jiang et al., 2020). Here in the current study, we identified a set of NFB learning-performance-related multimodal neuroimaging features, which greatly broaden our knowledge about the neural mechanisms of NFB learning effects. The results suggested that, in view of the underspecified and complex character of NFB training task, the individual difference in NFB learning performance is not attributed to single modality, the individual difference in SMR NFB performance is not attributed to one single factor, but modulated by the brain’s baseline neural activity, structure, function, and functional connections.

### Multimodal Predictors of SMR NFB Learning Index

#### EEG Predictors

According to existing findings, a well-known predictor of NFB learning index should be the baseline EEG activity. A higher baseline level of the training parameter (brain activity of the training target) is advantageous for better NFB training performance (Wan et al., 2014; Nan et al., 2015; Reichert et al., 2015). In consistence with previous studies (Reichert et al., 2015; Nan et al., 2018), we found that the eyes-open resting-state SMR power showed positive correlation with NFB learning index. Generally, the most relevant resting-state SMR power located in central regions, especially the left central regions. It has been proposed that a certain baseline level of SMR power is associated with a higher neural adaptability which may allow a better modulation of this EEG rhythm (Reichert et al., 2015).

#### fMRI Predictors

Similar to rest EEG, rsfMRI data are also easy to collect and have gained widespread applications. Two types of features, regional activities (ALFF/fALFF/ReHo) and FC of intrinsic brain networks, can be calculated from rsfMRI. However, there is no study to examined rsfMRI-based predictors for EEG NFB training performance. We found significant positive correlations between the NFB learning performance and the strength of a set of FC features, which were mainly observed within the cingulo-opercular network, between the sensorimotor network and occipital network. Similarly, FC having significant negative correlations with the learning performance were observed between the default mode network and occipital network.

Considering the underspecified content of NFB training task, successful learning performance requires participants to show self-initiated multitasking, such as to focus on inner mental thoughts and external stimuli at the same time. Therefore, while concentrating on real-time feedback, participants have to redirect attention away from irrelevant thoughts and toward goal-related thoughts (Kober et al., 2017). Hence, positive correlation between resting-state within-network FC of cingulo-opercular network might be explained by its positive effects for redirection of attention and maintenance of tonic alertness (Sadaghiani and D’Esposito, 2015). A previous study showed that the intention to control the moving bar of sham feedback is sufficient to engage a broad cingulate-opercular network related to cognitive control (Ninaus et al., 2013).

The default mode network is implicated in self-referential and integrative processes and it generally has negative FC with other task-positive networks during resting-state (Fox et al., 2005). The anticorrelation between the default mode network and task-positive visual networks may reflect the dichotomy between NFB training requiring introspectively oriented and extrospectively oriented attentional modes (Fransson, 2005). The strength of this negative correlation could be considered as an index of the degree of regulation in the default mode and task-positive networks, and showed positive correlation with behavioral performance (Kelly et al., 2008). Consistently, better NFB training performance showed negative correlation with resting-state FC between the default mode and occipital networks, i.e., the higher the negative correlation, the better the NFB training performance. Besides, performance during NFB training is also positively correlated with resting-state FC between occipital and sensorimotor networks. Both occipital and sensorimotor networks are known to support more specialized and mostly externally driven functions, and the FC between these two networks is normally low during resting-state (Doucet et al., 2011; Gu et al., 2015; Lee and Frangou, 2017). In addition to FC, we also observed positive correlation between ALFF and NFB learning performance in brain regions within the sensorimotor and occipital networks, such as the postcentral gyrus and lingual gyrus. Considering increased FC between the networks responsible for processing different types of sensory information, it can be inferred that better NFB learning performance might be related to multisensory integration.

#### sMRI Predictors

VBM analysis of sMRI data measures the neuroanatomy characteristic of the human brain, and it has been used in previous studies to investigate the link between brain anatomical properties and the individual difference in cognitive function (Kanai and Rees, 2011). In order to reveal the neuroanatomical basis of the ability to achieve self-regulation of one’s own brain activity, two studies investigated structural predictors of learning performance during up-regulation of SMR power (Ninaus et al., 2015; Kober et al., 2017). Compared with these two studies, the current study observed relevant findings on GMV and WMV of more widely distributed brain regions. Specifically, for the default mode network, a positive relationship between GMV and NFB performance was observed in the superior parietal gyrus, precuneus, and inferior temporal gyrus, and a negative relationship was observed in the frontal gyrus and temporal pole. Besides, a number of brain regions within the occipital network, including the middle occipital gyrus, calcarine sulcus, lingual gyrus, and fusiform gyrus, showed positive correlation between WMV and NFB performance. As mentioned before, NFB performance showed significantly correlation with the rsfMRI FC strength between the default mode network and occipital network. These results underscore the importance of these two functional networks to the capability of learning self-regulation of brain activity from the neuroanatomical perspective. So far, a very limited number of studies investigated structural predictors of NFB performance, and their results also explained the neuroanatomical basis for learning self-regulation of brain activity (Enriquez-Geppert et al., 2013; Ninaus et al., 2013, 2015; Kober et al., 2017). There are some differences between the results obtained in this study and those reported before, and the differences can be attributed to a number of reasons, such as different NFB protocols, training characteristics, and measurements of training performance. Future study should attempt to reveal a more general “NFB network” in the brain, regarding overlapping neuroanatomical correlates for different NFB protocols (Weber et al., 2020).

### Limitations

This present study has some limitations. First, the present study was constrained in terms of the sample size, which could limit and weaken the result of this study. Second, a corrected threshold was only used for extraction of EEG correlates. The results are sufficient to support our conclusion, but studies with more strict feature selection approach are needed to verify the results obtained here. Third, the multimodal prediction was primarily achieved by simple concatenating brain features from different modalities horizontally into a single, combined feature space, thus not allowing for a full use of the joint information among modalities (Sui et al., 2020). Forth, only within-session learning effect was evaluated in this study. There is no generally accepted best measure for assessing NFB learning success so far. One might speculate that observed predictors for NFB performance might be different if we had used another measure of learning performance. But here in this study, our main purpose is to validate the necessary of utilizing multimodal data when investigating predictors of NFB learning success, therefore we used a learning index (LI), which has been successfully applied in previous studies. Last but not least, we haven’t collected any behavioral data to characterize subject’s psychological state during NFB training, such as mental strategy. This should be considered in future study together with pre-NFB baseline factors to make a more comprehensive investigation of NFB learning performance predictors.

## Conclusion

In conclusion, inter-individual differences concerning the ability to regulate one’s brain activity are in the focus of current NFB research. Existing studies proposed that the individual differences in NFB learning performance can be attributed to electrophysiological and anatomical baseline characteristics. Our results support and extend these findings, since we found reliable predictors of within-session NFB learning performance from multimodal neuroimaging data. The results of this study highlight the importance of multimodal neuroimaging technique as a tool to explain the individual difference in learning performance during NFB training, and could provide a basic theoretical framework for development of individualization of NFB protocols.

## Data Availability Statement

The data supporting the findings of this study are available from the corresponding author upon reasonable request.

## Ethics Statement

The studies involving human participants were reviewed and approved by the Ethics Committee of Shenzhen University. The patients/participants provided their written informed consent to participate in this study.

## Author Contributions

ZZ, LR, and NY conceived and designed the experiments. LL, YW, YZ, and SH performed the experiments. LL and YW analyzed the data and wrote the main manuscript. GH and LZ verified the analytical methods. All authors reviewed the manuscript.

## Conflict of Interest

The authors declare that the research was conducted in the absence of any commercial or financial relationships that could be construed as a potential conflict of interest.

## Footnotes

1 https://github.com/NOCIONS/letswave6

2 http://www.neuro.uni-jena.de/cat/

3 https://www.fil.ion.ucl.ac.uk/spm/software/spm12/

4 http://warwick.ac.uk/snpm

5 http://rfmri.org/DPARSF

6 http://rfmri.org/dpabi
