# Supplementary material for: Multimodal Neuroimaging Predictors of Learning Performance of Sensorimotor Rhythm Up-Regulation Neurofeedback
Source: Front Neurosci. 2021 Jul 20;15:699999. doi: 10.3389/fnins.2021.699999 (PMC8329704; doi:10.3389/fnins.2021.699999)
Supplement: Supplementary file 1 [file Data_Sheet_1.docx]

**Supplementary Materials to “Multimodal neuroimaging predictors of learning performance of sensorimotor rhythm up-regulation neurofeedback”**

Linling Li^1,2,3^**^†^**, Yinxue Wang^1,2,3^**^†^**, Yixuan Zeng^6^, Shaohui Hou^1,2,3^, Gan Huang^1,2,3^, Li Zhang^1,2,3^, Nan Yan^5^, Lijie Ren^6#^, Zhiguo Zhang^1,2,3,4#^

^1^School of Biomedical Engineering, Health Science Center, Shenzhen University, Shenzhen, China

^2^Guangdong Provincial Key Laboratory of Biomedical Measurements and Ultrasound Imaging, Shenzhen, China

^3^Marshall Laboratory of Biomedical Engineering, Shenzhen University, Shenzhen 518060, China

^4^Peng Cheng Laboratory, Shenzhen, China

^5^CAS Key Laboratory of Human-Machine Intelligence-Synergy Systems, Shenzhen Institutes of Advanced Technology, Chinese Academy of Sciences, Shenzhen, China

^6^Department of Neurology, the First Affiliated Hospital of Shenzhen University, Shenzhen Second People's Hospital, Shenzhen, China

**^†^**These authors have contributed equally to this work.

**^#^Correspondence**

Zhiguo Zhang [zgzhang@szu.edu.cn](mailto:zgzhang@szu.edu.cn)

Lijie Ren 13631605966@126.com

| **Supplementary Table S1.** List of brain regions with grey/white volumes which are significantly correlated with the NFB learning index LI (P < 0.005, Cluster size>50). | | | | | | | | |
| --- | --- | --- | --- | --- | --- | --- | --- | --- |
| **Region** |  | **BA** | **MNI coordinates** | | | **Cluster size** | **T-value** | **P-value** |
|  |  |  | **X** | **Y** | **Z** |  |  |  |
| **Gray Matter, positive correlation** |  |  |  |  |  |  |  |  |
| Superior Parietal Gyrus (SPG) | L | 7 | -21 | -65 | 66 | 215 | 4.33 | 0.0005 |
| Inferior Temporal Gyrus (ITG) | L | 20 | -51 | -51 | -11 | 76 | 3.90 | 0.0008 |
| **Gray Matter, negative correlation** |  |  |  |  |  |  |  |  |
| Superior Frontal Gyrus (SFG) | R | 8 | 24 | 36 | 54 | 59 | 3.67 | 0.0002 |
| Middle Frontal Gyrus (MFG) | L | 8 | -29 | 11 | 51 | 173 | 3.46 | 0.0013 |
| Supramarginal Gyrus (SMG) | L | 48 | -54 | -26 | 27 | 62 | 3.69 | 0.0009 |
| Middle Temporal Gyrus (MTG) | R | 21 | 53 | 2 | -18 | 203 | 3.71 | 0.0006 |
| Middle Temporal Pole (MTP） | L | 38 | -44 | 18 | -36 | 106 | 3.71 | 0.0005 |
| **White Matter, positive correlation** |  |  |  |  |  |  |  |  |
| Precuneus | L | NA | -15 | -56 | 69 | 172 | 4.25 | 0.0003 |
| Precuneus | L | NA | -5 | -78 | 50 | 155 | 6.13 | 0.0001 |
| Supplementary Motor Area (SMA) | R | NA | 5 | -12 | 60 | 260 | 4.4 | 0.0001 |
| Medial Frontal Gyrus | R | NA | 12 | 56 | 36 | 58 | 3.06 | 0.0026 |
| Middle Occipital Gyrus (MOG) | L | NA | -33 | -83 | 11 | 80 | 4.09 | 0.0013 |
| Calcarine | L | NA | -6 | -93 | 9 | 146 | 4.13 | 0.0007 |
| Lingual Gyrus | R | NA | 8 | -69 | 5 | 68 | 3.25 | 0.0035 |
| Fusiform Gyrus (FG) | L | NA | -29 | -56 | -15 | 281 | 4.96 | 0.0002 |
| Middle Temporal Pole (MTP) | R | NA | 35 | 14 | -41 | 63 | 3.39 | 0.0035 |
| **White Matter, negative correlation** |  |  |  |  |  |  |  |  |
| Hippocampus | R | NA | 35 | -15 | -11 | 239 | 3.52 | 0.0006 |
| Precentral Gyrus (PCG) | L | NA | -26 | -9 | 45 | 140 | 4.06 | 0.0005 |

| **Supplementary Table S2.** List of brain regions with rsfMRI ALFF which are significantly correlated with the NFB learning index LI (P < 0.005, Cluster size>50). | | | | | | | | |
| --- | --- | --- | --- | --- | --- | --- | --- | --- |
| **Region** |  | **BA** | **MNI coordinates** | | | **Cluster size** | **T-value** | **P-value** |
|  |  |  | **X** | **Y** | **Z** |  |  |  |
| **Postcentral Gyrus (PCG)** | L | 4 | -33 | -24 | 51 | 51 | 5.01 | 0.0009 |
| **Lingual Gyrus (LG)** | R | 27 | 12 | -30 | -6 | 66 | 6.49 | 0.0002 |
| **Hippocampus** | R | 20 | 30 | -15 | -18 | 66 | 4.85 | 0.0003 |
| X, Y, Z are peak MNI coordinates of activated clusters; MNI, Montreal Neurological Institute. BA, Broadmann’s area; NA, not available; PCG, Postcentral Gyrus; LG, Lingual Gyrus. | | | | | | | | |

| **Supplementary Table S3.** Correlation, significance and mean absolute error (MAE) of LI prediction results using different models. | | | |
| --- | --- | --- | --- |
| **Region** | **R-value** | **P-value** | **MAE** |
|  |  |  |  |
| **Linear Regression (LR)** |  |  |  |
| EEG | 0.5519 | 0.0028 | 0.6136 |
| sMRI | 0.6577 | 0.0002 | 0.6414 |
| ALFF | 0.6227 | 0.0005 | 0.6414 |
| rsfMRI FC | 0.4774 | 0.0118 | 0.7870 |
| Multimodal | 0.8101 | <0.0001 | 0.4403 |
| **Automatic Relevance Determination Regression (ARDR)** |  |  |  |
| EEG | 0.5519 | 0.0028 | 0.6136 |
| sMRI | 0.4912 | 0.0093 | 0.8615 |
| ALFF | 0.7335 | <0.0001 | 0.5625 |
| rsfMRI FC | 0.3626 | 0.0630 | 0.9722 |
| Multimodal | 0.7997 | <0.0001 | 0.5007 |
| **Random Forest Regressor (RFR)** |  |  |  |
| EEG | 0.5519 | 0.0028 | 0.6136 |
| sMRI | 0.4534 | 0.0175 | 0.6679 |
| ALFF | 0.7742 | <0.0001 | 0.5432 |
| rsfMRI FC | 0.6450 | 0.0003 | 0.5815 |
| Multimodal | 0.7982 | <0.0001 | 0.5651 |


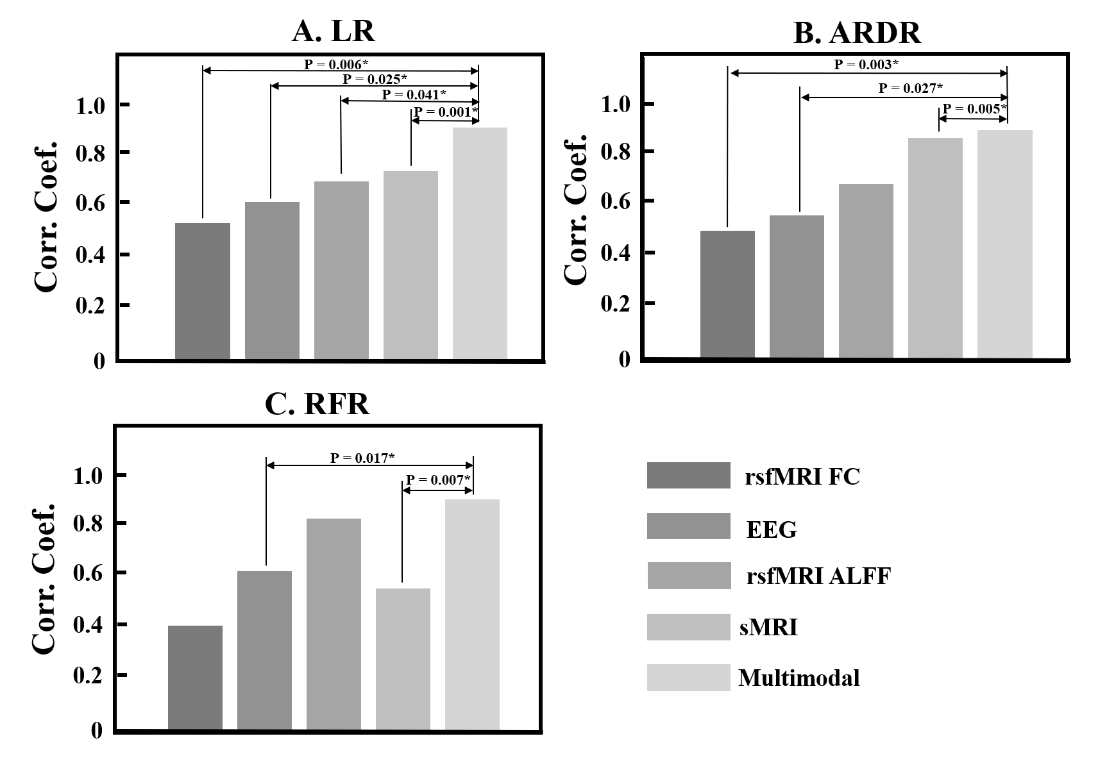


**Supplementary Figure S1.** The comparison results of correlation coefficients between predicted and actual LI values among five different feature sets using different models (LR: linear regression; ARDR: automatic relevance determination regression; RFR: random forest regressor).
